# Deciphering the Patterns of Genetic Admixture and Diversity in the Ecuadorian Creole Chicken

**DOI:** 10.3390/ani9090670

**Published:** 2019-09-11

**Authors:** Paula Alexandra Toalombo Vargas, José Manuel León, Luis Rafael Fiallos Ortega, Amparo Martinez, Alex Arturo Villafuerte Gavilanes, Juan Vicente Delgado, Vincenzo Landi

**Affiliations:** 1 Escuela Superior Politécnica de Chimborazo, Panamericana Sur Km 1 ½, EC060155, Riobamba, Ecuador; paulasol37@yahoo.es (P.A.T.V.); luisfior@yahoo.es (L.R.F.O.); dralexvillafuerte@hotmail.com (A.A.V.G.); 2 Centro Agropecuario Provincial de Córdoba, Diputación Provincial de Córdoba, 14071 Córdoba, Spain; jomalejur@yahoo.es; 3 Department of Genetics, Faculty of Veterinary Sciences, University of Cordoba, 14071 Cordoba, Spain; amparomartinezuco@gmail.com (A.M.); id1debej@uco.es (J.V.D.); 4 Animal Breeding Consulting sl., C/. Astrónoma Cecilia Payne, ID-1, 8-PE, Rabanales 21, 14014 Cordoba, Spain

**Keywords:** Creole chicken, microsatellites markers, D-loop, conservation

## Abstract

**Simple Summary:**

In Ecuador, the production of Ecuadorian Creole chicken is of crucial importance in the economy and for the nutrition of families. These chickens represent a focal point in scientific research for three main reasons: (1) they are an unknown genetic resource derived from 500 years of environmental and human selection and represent an important reservoir of genetic variability and adaptability; (2) Ecuadorian Creole chicken production is normally familiar, in a marginal dimension, and it is an important source of economic input for medium–low income communities; and (3) being a local genetic resource, it is available to local communities without intermediary international enterprises and represents the starting point for food sovereignty. We aimed to measure the level of genetic diversity and its phylogenetic position compared with other outgroup breeds using information from microsatellite and mitochondrial markers. Our results showed that these chicken populations represent a great reservoir of genetic variability; however, the genetic fragmentation owing to the high geographical diversity of the country could compromise the conservation status and, therefore, the establishment of an official breeding program is needed for the conservation and valuation of these avian populations, with this genetic characterization being a first step.

**Abstract:**

Latin American Creole chickens are generally not characterized; this is the case in Ecuador, where the lack of scientific information is contributing to their extinction. Here, we developed a characterization of the genetic resources of Ecuadorian chickens located in three continental agroecosystems (Pacific coastal, Andean, and Amazonian). Blood samples of 234 unrelated animals were collected in six provinces across Ecuador: Bolívar, Chimborazo, Cotopaxi, Guayas, Morona Santiago, and Tungurahua, in order to perform a genetic characterization and population structure assessment using the AVIANDIV project microsatellites panel (30 loci) and D-loop sequences of mitochondrial DNA and comparing with reference data from other breeds or genetic lines. The results indicate that Ecuadorian Creole chickens are the result of the admixture of different genetic groups that occurred during the last five centuries. While the influence of South Spanish breeds is demonstrated in the colonial age, genetic relationships with other breeds (Leghorn, Spanish fighter cock) cannot be discarded. The geographical configuration of the country and extreme climate variability have influenced the genetic isolation of groups constituting a homogeneous genetic status into the whole population. This is not only a source of genetic variation, but also a critical point because genetic drift produces a loss of genetic variants.

## 1. Introduction

In Ecuador, the Creole chicken is an important genetic resource utilized in backyard productions, and is part of the traditional diet and an important economic resource for families. The meat and eggs of these birds have a high nutritional value accessible to the entire population owing to its low purchasing power. According to the National Finance Corporation, in 2016, after conducting an economic analysis of poultry production in Ecuador, it was indicated that between 2013 and 2016, the raising of field poultry decreased by 27%, while an increase in the industrial rearing system was observed, drawing attention to the need for the study and characterization of the Creole chicken population [1].

The origin of the Creole chicken on the American continent is still controversial; current Creole chickens derive from the introduction of European animals during the Spanish colonization in the 15th century [2]. According to other authors, however, the chicken already existed in pre-Columbian civilization. This theory would be confirmed by one record in which Francisco Pizarro describes the presence of the chicken in previously unknown indigenous settlements and by the presence, over the entire South American continent, of animals producing blue-colored eggs, typical of the Asian germplasm [3].

The arrival of chickens from Polynesia is supported by different hypotheses. According to some authors, the relative geographic proximity between Chile and the archipelagos of the Pacific could justify some type of commerce [4,5,6].

It is probably that the current population is, therefore, the result of different admixture events, including the introduction of the industrial genetic types in recent times, as can be observed by the extreme phenotypic variability.

These populations represent an important example of adaptation to different climates. In fact, Ecuador presents drastically changing climatic and orographic conditions (Alpine in the Andean region, tropical in the eastern region, and subtropical in the coastal area) within a few kilometers, ranging from a few meters above sea level with very high levels of temperature and moisture to the central region at more than 4000 meters above sea level with a cold and dry environment.

The adaptation of the local populations of chickens to these extreme environmental conditions on the one hand ensures a source of protein in the nutrition of the family and, on the other, offers a source of genetic richness to develop the new trends in sustainable free-range avian production systems, respecting the welfare of animals.

Currently, these animals are at a permanent risk of extinction because of the lack of serious scientific studies regarding their characterization, which could support their general recognition. According to Alderson [7], the knowledge of the characteristics of populations is the first step in conservation programs. The absence of information about the status of genetic variability along with the absence of governmental or private breeding plans, as well as the introduction of industrial genetic types in the country, are critical factors that threaten these traditional populations. This situation is similar to that described in other countries in the region [8]. In the present study, we used two genetic tools widely employed in the methodology of investigating the genetic variability and the genetic relationship among populations in several species [9,10,11,12,13]. First, 30 microsatellite markers were used to determine the population structure and variability, after which the sequence of the D-loop region of mitochondrial DNA was used to identify different lineages that could demonstrate the hypothesis of multiple origins of the Ecuadorian chickens with a view to develop the basic framework for the conservation, valuation, and general recognition of these populations.

## 2. Materials and Methods 

### 2.1. Ethics Statement

Ethical approval was not needed for this study. Blood samples from Ecuadorian chickens were collected by qualified veterinarians during their routine practice within the framework of official programs aimed at the identification, monitoring of health, and parentage confirmation of the breeds and populations included in our study. In terms of collection, the fieldwork did not involve any endangered or protected species. Hair roots were manually collected without any injury in the back of the animals. No other kind of tissues (blood, meat, or other) were used in this study. The other breeds are data proceeded from another study inside our research group.

### 2.2. Sampling and DNA Extraction

A total of 244 unrelated animals of both sexes were randomly sampled from six provinces representing all the continental agroecosystems of Ecuador: Bolívar, Chimborazo, Cotopaxi, Guayas, Morona Santiago, and Tungurahua (Supplementary Material: Figure S1, Table S1). Specific sampling and geospatial locations are reported in Table S1, according to the 26 municipalities within the 6 provinces of precedence. Up to 200 μL of EDTA K3 (BD Vacutainer, Franklin Lakes, NJ, USA) anticoagulated blood was loaded in the collection circle of Whatman^®^ FTA^®^ Cards (GE healthcare Life Science, Little Chalfont, Buckinghamshire, UK). The cards were dried at 25 °C for a minimum of 3 h and then stored in paper envelopes until use. DNA was extracted for both mitochondrial DNA and STRs (short tandem repeats) analysis, following a modification of the method described by Walsh et al. [14]. Briefly, three circles were cut in the spotted cards using a 2 mm Harris Micro punch (GE healthcare Life Science, Little Chalfont, Buckinghamshire, UK), which was cleaned using 1% bleach solution between each sample. The circles were placed in a PCR plate and incubated in 100 µL of a 5% CHELEX 100 resin solution (Bio-Rad, Hercules, CA, USA) at 95 °C for 15 min, 60 °C for 15 min, and finally 99 °C for 3 min. The lysate was removed and frozen at −20 °C until use.

### 2.3. Molecular Marker Analysis

#### 2.3.1. Mitochondrial DNA D-Loop Analysis

The mitochondrial control region amplification was performed from nucleotide position 16,741 to 1280 of the complete mitochondrial DNA sequence (accession number NC007235) [15], including a part of the hypervariable region of the chicken mitochondrial genome (from nucleotide 1 to 1232). Oligos used have been described in Ceccobelli et al. [16].

The PCR was performed in a total volume of 25 µL as follows: PCR buffer (Aidlab Biotechnologies Ltd., Beijing, China) 1× (75 mM Tris-HCl pH 9, 25 mM KCL, 2 mM MgCl_2_), 200 µM of each dNTP (deoxyribonucleotide triphosphate) (Thermo Fisher Scientific, Waltham, MA, USA), 0.2 µM of each oligonucleotide, 1 U of Taq polymerase (Aidlab Biotechnologies Ltd., Beijing, China), and about 30 ng of DNA. The reaction mixtures were placed in a thermocycler Bio-Rad C1000 touch (Hercules, CA, USA) under the following amplification conditions: 95 °C for 5 min, followed by 34 cycles at 95 °C for 45 s, 59 °C for 1 min, and 72 °C for 2 min, with a final extension at 72 °C for 45 min.

Amplicons’ quality and consistency were assessed in a 1% agarose gel stained with ethidium bromide and, finally, 5 µL was incubated with one unit of FastAP Thermosensitive Alkaline Phosphatase (Thermo Fisher Scientific, Waltham, MA, USA) and 10 units of Exonuclease I enzyme (Thermo Fisher Scientific, Waltham, MA, USA) at 37 °C for 15 min and 80 °C for 15 min. Sequencing reactions were performed in both directions using PCR oligos at Macrogen Inc. services (Madrid, Spain).

Nucleotide sequences were checked and edited using MEGA 5.0 [17]. A fragment of 506 base pairs (from nucleotide 1 to 505) was used in the analysis. The sequences have been submitted to the NCBI database with accession numbers from MK649380 to MK649613.

#### 2.3.2. Microsatellites Markers

We used a total of 30 microsatellite markers from the AVIANDIV project (https://aviandiv.fli.de/). Technical characteristics such as PCR and electrophoresis conditions were followed as reported in Ceccobelli et al. [16]. Genotypes were read with ABI PRISM GeneScan 3.1.2 (Applied Biosystems, Forster City, CA, USA) and interpreted with ABI PRISM Genotyper 3.7 NT (Applied Biosystems, Forster City, CA, USA).

### 2.4. Statistical and Genetic Analyses 

#### 2.4.1. Mitochondrial DNA D-Loop

Sequence editing, alignment, and construction of data matrices were performed with Mega 5.0 [17] and gBlocks 0.91b [18]. For statistical analysis, the samples were organized into the six provinces (Table S1): Bolívar, Chimborazo, Guayas, Tungurahua, Cotopaxi, and Morona Santiago.

The number of haplotypes, polymorphic sites, and nucleotides and haplotype diversity estimates for the domesticated and wild populations were calculated using DnaSP v5 (http://www.ub.edu/dnasp/) [19]. Analysis of molecular variance (AMOVA) [20] was used to calculate genetic variation and genetic differentiation between populations by performing 10,000 permutations, using different clustering and taking into account the geographical distribution ARLEQUIN v3.1 (http://cmpg.unibe.ch/software/arlequin35/) [21].

In addition, computed pairwise comparisons of FST (fixation index of subpopulation total) values with 1000 permutations were obtained using ARLEQUIN v3.1 (http://cmpg.unibe.ch/software/arlequin35/) [21].

To establish phylogenetic relationships between haplotypes and their frequencies, a haplotype network was constructed using the median-joining method [22] with NETWORK v4.6.0.0 (www.fluxus-engineering.com) and setting default parameters. The relationships between haplotypes were also analyzed using phylogenetic inference. The final dataset included all sequences analyzed in this study and those retrieved from the NCBI database, representing the main clades described in the literature: AF512092 (haplogroup A), AF512125 (haplogroup B), AF512263 (haplogroup C), AB007726 (haplogroup D), AB114061 (haplogroup E), GU448405 (haplogroup F), GU448894 (haplogroup G), and GU448603 (haplogroup K).

#### 2.4.2. Microsatellites Markers

For microsatellites statistical analysis, a dataset was constructed considering 15 chicken breeds for population relationship and geneflow assessment, including, as outgroups, nine breeds from Spain (including the Balearic archipelago), one from Africa, one Creole breed from Chile, and finally three commercial lines (Supplementary Material, Table S2). ECU: Ecuadorian Creole Chicken; AAZ: Andaluza Azul; CASN: Castellana Negra; CES: Combatiente Español; EAZ: Extremeña Azul; IB: Ibicenca; MLL: Mallorquina; PPA: Pita Pinta; SUR: Sureña, UP: Utrerana Perdiz; ARAU: Araucana; BRAH: Brahma; NIG: Nigeria; CORN: Cornish; LEGH: Leghorn. Statistical analysis of the Ecuadorian samples was also performed, considering each sampling location independently (Table S1) to study the internal diversity of that population.

The mean number of alleles per locus and population and observed and expected heterozygosity estimates were calculated using the EXCEL MICROSATELLITE TOOLKIT 3.1.1 (http://animalgenomics.ucd.ie/sdepark/ms-toolkit/) [23]. Effective allele number (AE) by marker and by population was calculated using POPGENE v1.32 (https://sites.ualberta.ca/~fyeh/popgene.html) [24]. In the Ecuadorian samples organized by province, we estimated private allele richness with HP-Rare v 1(http://www.montana.edu/kalinowski/software/hp-rare.html) [25] using the reference number of individuals as 10. This is a parameter estimating the private (exclusive) allele number corrected by the sample size, and is an indicator of differentiation in a reduced group of individuals [26]. Deviation from the Hardy–Weinberg equilibrium (HWE) at each locus within populations was tested using GENEPOP 4.0 (http://genepop.curtin.edu.au/) [27], applying the Fisher’s exact test and using the Markov chain algorithm with default settings. *p*-values [28] were calculated and corrected for multiple tests using the Bonferroni method [29]. The FIS, FIT, and FST (fixation index of individual, total population, and subpopulation, respectively) coefficients [30] and factorial correspondence analysis (AFC) was calculated using Genetix v.4.05.2 (https://kimura.univ-montp2.fr/genetix/) [31].

Reynolds distances [32], showing genetics divergence among breeds, were estimated using POPULATIONS v1.2.28 (http://bioinformatics.org/populations/) [33], and a neighbor-net was constructed as implemented in SplitsTree4, in order to represent the relationships between breeds graphically and to depict evidence of admixture [34].

The pairwise FST matrix calculation and graphical representation and AMOVA were performed using Arlequin v.3.5 (http://cmpg.unibe.ch/software/arlequin35/) [21].

The genetic structure and geneflow were assessed using STRUCTURE v.2.3.4 [35]. The software was first run on a dataset including all the breeds used for comparison and considering the Ecuadorian samples as a unique population using the admixture model and correlated allele frequencies (burning-in 300K and iteration 600K); secondly, it was run using only the Ecuadorian samples, organized according to sampling location (municipality; Supplementary Material, Table S1 and Figure S6), and applying the prior model parameter *locprior* to the population model [36]. Each run was repeated 20 times, after which it was submitted to CLUMPACK (http://clumpak.tau.ac.il/) to perform matrix averaging and obtain individual bar plot representation [37]. The most likely number of K calculations was performed using the STRUCTURE HARVESTER web server (http://taylor0.biology.ucla.edu/structureHarvester/) using both the method described by Evanno et al. and the log probability plot, and is reported in Figure S4 and S5 (Supplementary Material), respectively [38,39,40]. R 3.6.1 was used to display spatial interpolations of the individual membership coefficient using geographical coordinates recorded from Google Earth 7.3.1.4507 according to the municipality data (Supplementary Material, Table S1) [41,42].

## 3. Results

### 3.1. Mitochondrial DNA Phylogeny

We analyzed 244 DNA samples, and 234 mitochondrial DNA sequences were obtained and characterized, which comprised a length of 850 bp that included the central hypervariable region of the control region. The six populations were polymorphic, with several haplotypes ranging from 4 (Tungurahua) to 10 (Morona Santiago). A total of 24 haplotypes with 123 polymorphic sites were identified. The average haplotypic diversity was 0.359, ranging from 0.461 (Morona Santiago) to 0.292 (Cotopaxi). The average nucleotide diversity was 0.00547 (Table 1) and ranged from 0.00091 (Bolivar) to 0.01121 (Tungurahua).

**Figure 1:**
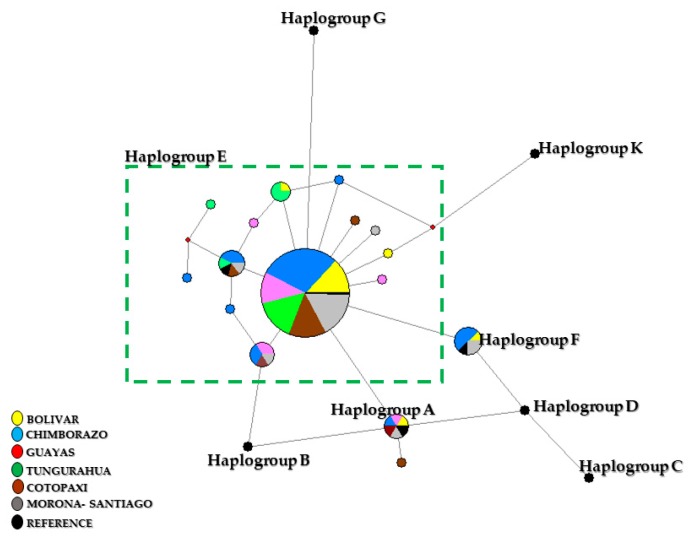
Median-joining network for the Ecuadorian Creole chicken breed compared with reference sequences from Ceccobelli et al. [9]. Circled areas are proportional to the haplotype frequencies and colors identify the province of the samples.

The median-joining network (Figure 1) was constructed with eight reference sequences, where 21 haplotypes were obtained. The most frequent haplotype in the entire population was H5. Regarding the absolute frequencies in the different provinces, Bolivar presented the haplotypes H1, H5, H6, H10, and H11; Chimborazo H1, H5, H6, H12, H13, H15, H17, and H18; Guayas H1, H5, H14, H15, and H16; Tungurahua H5, H10, H18, H20, and H21; Cotopaxi H1, H5, H15, H18, H20, and H21; and Morona Santiago H1, H5, H6, H9, H15, and H18. Comparing all the sequences with the reference haplotypes, 83.76% of the samples were grouped with a main haplogroup E, with 26, 58, 22, 30, 27, and 33 samples from Bolivar, Chimborazo, Guayas, Tungurahua, Cotopaxi, and Morona Santiago, respectively; while in haplogroup F, 2.98% of the samples were clustered, in which 1, 6, 2, 1, and 3 belonged to Bolívar, Chimborazo, Guayas, Cotopaxi and Morona Santiago, respectively; and finally, in haplogroup A, 2.55% of the samples were clustered with one sample each from Guayas, Cotopaxi, and Morona Santiago (Figure 1).

In the AMOVA calculation based on the polymorphism of the D-loop sequence, tree comparisons were made (Supplementary Material, Table S3), showing negative variation in the first and last groupings (*p* < 0.05), whereas when Morona Santiago was compared with the rest of the provinces, a highly positive percentage of variation was obtained (0.24, *p* < 0.05). 

### 3.2. Microsatellites Markers

#### 3.2.1. Marker Polymorphism and Diversity

All the microsatellites used were polymorphic, finding a minimum of three (MCW098, MCW103) and a maximum of 19 (LEI0234) alleles, with a mean 8.1 ± 4.21 number of alleles of (Supplementary Material, Table S4). AE ranged from 1.50 of MCW014 to 6.67 of LEI0234 with the mean general value of 3.17 ± 1.30.

The highest expected heterozygosity was found for LEI0234 with a value of 0.852 and the lowest for MCW014 with a value of 0.331; the mean value of the whole dataset was 0.637 ± 0.14 (Table S4). The observed heterozygosity values ranged from a maximum of 0.788 for the LEI0234 marker and a minimum of 0.091 for the MCW014, with a mean value of 0.544 ± 0.144. In this study, most loci (23) had high PIC (polymorphic information content) values (PIC > 0.5), so they could be defined as very informative, with the exception of MCW014, MCW248, MCW103, MCW222, MCW098, MCW03, and MCW216 (PIC < 0.5), which were classified as moderately informative (Table S4).

FIS index indicates the excess of homozygosity in a subpopulation and, with reference to molecular markers, informs if a particular pattern of reduction in diversity owing to several causes exists. In this study, FIS ranged from a minimum of −0.034 (MCW014) to a maximum of 0.727 (MCW014) with an average of 0.146 (0.1254–0.1638).

Genetic diversity parameters in the dataset including the 15 breeds used for the population study comparison and genetic relationship assessment are shown in Table S5 (Supplementary Material). The mean number of alleles was 4.89 ± 2.62, ranging from a maximum of 7.61 in ECU to 3.04 in LEGH, but as a better estimation, we reported the AE, which is a parameter that performs a correction according to the number of samples. In this case, the value again ranged from 3.85 in ARAU to 2.13 in LEGH. Expected and observed heterozygosity were low (0.546 ± 0.357 and 0.491 ± 0.014, respectively), while the FIS index, as was expected in a dataset including very divergent breeds, showed a clear excess of homozygosity with a mean value of 0.113, which was positive and significant (*p* < 005), as were the results of all the populations with the exception of MLL, which registered a value of –0.016 (generally assumed as zero, and thus no deviation in expected homozygosity).

Examining the diversity of the 30 microsatellite markers within the 244 animals in the 6 provinces of Ecuador (Table 2), an average number of alleles per locus of 5.67 ± 0.32 was found, ranging from 5.37 alleles in Tungurahua to 6.20 in Chimborazo. The AE and private allelic richness ranged from a minimum of 3.39 and 0.19 (Cotopaxi) to a maximum of 3.60 and 0.31 (Morona Santiago and Tungurahua), respectively.

The mean heterozygosity expected varied from 0.60 (Cotopaxi) to 0.65 (Guayas), while the heterozygosity observed varied from 0.52 (Cotopaxi) to 0.590 (Guayas), (Table 2). The FIS index showed the lowest value of 0.097 for Morona Santiago and the highest of 0.138 for Tungurahua (*p* < 0.05).

In all the 180 HWE tests (30 loci in 6 provinces, Supplementary Material, Table S6), significant deviations from the HWE were observed (*p* < 0.05) in 56 cases (31.11%). Most specifically, an imbalance was found in 6, 5, 4, and 3 provinces in MCW014 and LEI0192; MCW330; MCW104; and MCW034, MCW078, MCW123, and MCW165, respectively. Chimborazo was the population with the most unbalanced loci (15), while Morona Santiago was the one with the least (6).

#### 3.2.2. Population Structure

**Figure 2:**
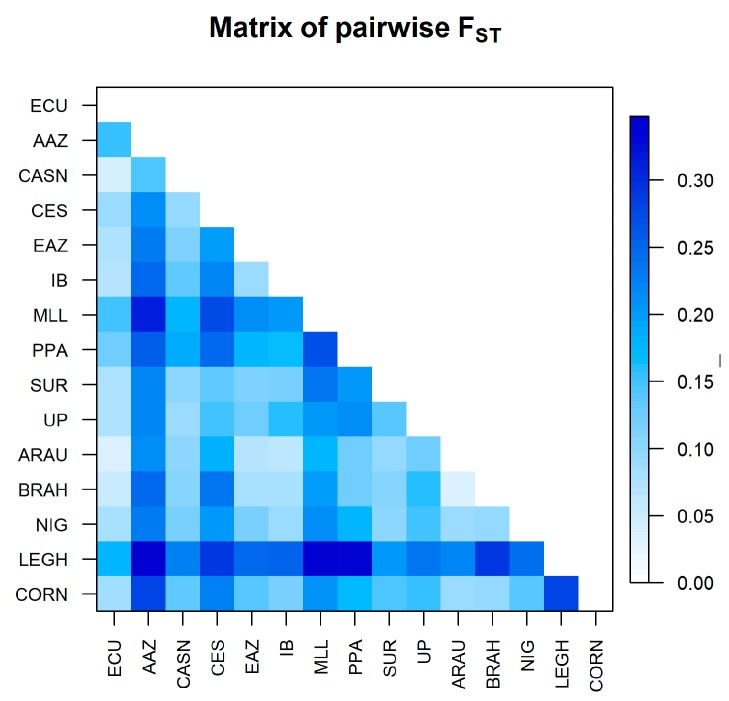
Pairwise fixation index of subpopulation total (FST) distance matrix. ECU: Ecuadorian Creole Chicken; AAZ: Andaluza Azul; CASN: Castellana Negra; CES: Combatiente Español; EAZ: Extremeña Azul; IB: Ibicenca; MLL: Mallorquina; PPA: Pita Pinta; SUR: Sureña, UP: Utrerana Perdiz; ARAU: Araucana; BRAH: Brahma; NIG: Nigeria; CORN: Cornish; LEGH: Leghorn.

Reynold and FST distance values are shown in Table S7 (Supplementary Material, Table S2). The breeds with the lowest FST with respect to Ecuadorian chickens are CASN and ARAU (0.042 and 0.041, respectively), while the Leghorn is the one with the highest FST (0.212). The Reynolds distance indicated the highest value with LEGH (0.193) and the lowest against CASN and ARAU (0.043 and 0.042, respectively). FST distances are represented with a gradient graphic (Figure 2) and confirm these findings. 

**Figure 3:**
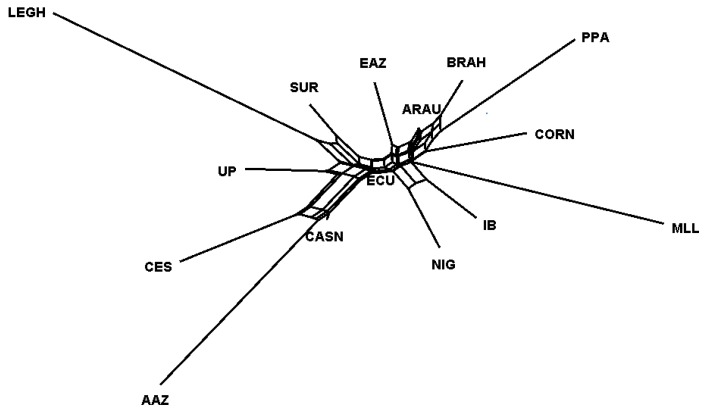
Neighbor-net dendrogram constructed using Reynolds distance among 15 chicken breeds. ECU: Ecuadorian Creole Chicken; AAZ: Andaluza Azul; CASN: Castellana Negra; CES: Combatiente Español; EAZ: Extremeña Azul; IB: Ibicenca; MLL: Mallorquina; PPA: Pita Pinta; SUR: Sureña, UP: Utrerana Perdiz; ARAU: Araucana; BRAH: Brahma; NIG: Nigeria; CORN: Cornish; LEGH: Leghorn.

Therefore, in the same figure, we can observe a lower mean value of FST (about <0.1) of the Ecuadorian chicken not only against the IB, EAZ, CES, and CASN, a group of Spanish breeds, but also with NIG, BRAH, ARAU, and CORN. AAZ, LEGH, and MLL showed higher values of FST (about >0.3). Neighbor-net representing the Reynolds distance confirmed these findings; ECU clustered in an intermediate position between the Spanish breeds CASN, SUR, and UP and another branch formed by ARAU, BRAH, PPA, CORN, and MLL. We can observe the nearest position at the center of the admixture network, indicating a lower genetic distance of ARAU with the cluster comprising CES, AAZ, and CASN (Figure 3).

Figure S2 (Supplementary Material) shows the factorial analysis of correspondence of the Ecuadorian chicken breeds compared with other populations (Table S2). With the first axis of the AFC, LEGH can be differentiated from the rest of the breeds; in the second axis, PPA, AAZ, and ECU are differentiated; while with the third axis, it is possible to differentiate only MLL. ECU differs from the rest of the populations included in the study, showing a shorter differentiation from ARAU and BRAH chicken breeds.

The graph derived from STRUCTURE shows the analysis of the genetic structure of the Ecuadorian population and the other breeds used for comparison in this study. When K = 2, the individuals are grouped in two clusters; one includes AAZ, MLL, UP, and LEGH (yellow), while the second cluster (blue) grouped the rest of the populations (Supplementary Material, Figure S3). The animals of the present study seem to be divided between the two clusters without any kind of preferential structure. This behavior is repeated almost until K5, where it can be clearly observed that in ECU, there are individuals with genomes of different proportions. According to the ln likelihood plot for each K (Figure S4), we may infer that the most likely K was 11. In K11, almost all the populations appear to be homogeneously separated; the Ecuadorian individuals of the present study form a homogeneous cluster, although it is possible to detect a group closely related to SUR and another one with CES. This can be seen clearly in the table of Q values, where ECU appears to be assigned 15% to the CES cluster and 11.2% to that of SUR (Supplementary Material, Table S8).

**Figure 4:**
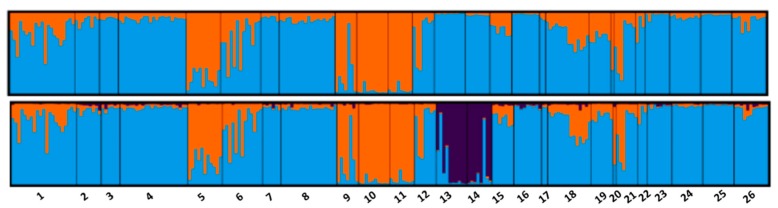
Bar plot of K2 and K3 results (individual membership coefficient) of STRUCTURE using the *locprior* assumption divides according to the municipality of sampling. 1, Echeandia; 2, San Pablo; 3, Chimbo; 4, Columbe; 5, Bucay; 6, San Vicente-Cumanda; 7, Pallatanga; 8–9, Nabuzo-Penipe; 10, Licto; 11, Chambo; 12, Guano; 13, Pelileo; 14, Tisaleo; 15, Ambato; 16, Baños; 17, Santa Cecilia; 18, Pujili; 19, Poalo; 20, Belisario; 21, Salcedo; 22, Saquisili; 23, Sevilla Don Bosco; 24, Sinai; 25, Tres Marias; 26, Sevilla De Oro.

**Figure 5:**
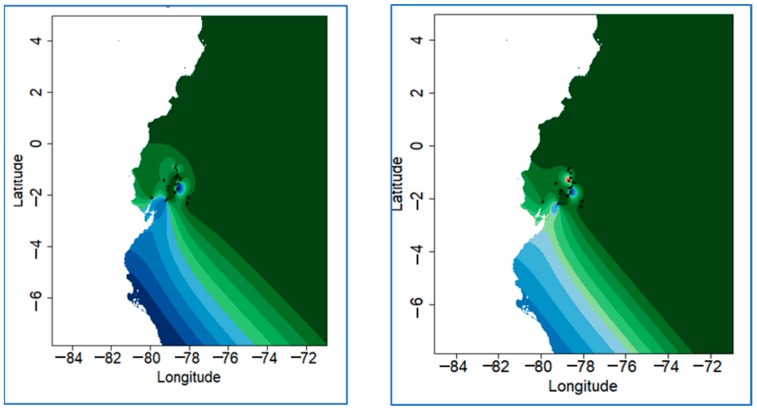
Plot of STRUCTURE Q matrix formed by K vectors of ancestry coefficients within a geographic map using an ascii raster file of the study area. Figure a (right), K2; and Figure b (left), K3.

Examining the result of the Bayesian analysis considering only the Ecuadorian samples and the geographical coordinate of the samples corresponding to the 26 municipalities (Table S1), we can observe a certain stratification within the population. In Figure 4, K2 and K3 are depicted. Most likely K, as in the general STRUCTURE calculation, is not consistent according to the Evanno method, even if several high peaks are reported in K3, K7, K20, and K22 (Figure S5). In K2, it can be seen that the municipalities of Bucay, Penipe-Nabuzo, Chambo, and Licto clustered in a homogeneous group (average Q 87%, supplementary material: Table S9), although some individuals in other municipalities seem to share this proportion. In K3, we found a third well-delimited cluster (average Q 63%), which comprised the municipalities of Pelileo and Tisaleo. The graphical representation of the assignment values of STRUCTURE plotted on the map (Figure 5) shows, as in K2, that the two points in the map belonging to Bucay, Penipe-Nabuzo, Chambo, and Licto (Figure S6) are located on the mountain side region and by a municipality (Bucay) that is very distant on the plains in the tropical zone.

The map representing K3 shows a new group corresponding to the valley that hosts the city of Ambato, in the center of the mountain area (Pelileo and Tisaleo).

## 4. Discussion

### 4.1. Mitochondrial DNA D-loop Analysis 

Ecuadorian chicken breeds are of great importance in the economy and for the nutrition of families within the country, and they are an important source of genetic richness. Even so, all these populations are in extreme danger of extinction owing to a severe reduction in population, uncontrolled genetic migrations from industrial breeds, and especially the lack of recognition by the administration and the society.

In this study, we present the first results of genetic diversity in Ecuadorian chicken breeds following two different approaches. Mitochondrial DNA polymorphism was used to understand the evolution of the breeds, and the population structure and diversity were investigated based on genome composition and microsatellites. The aim was to augment the meager scientific knowledge existing about these populations with genetic characterization as the first step toward a conservation program [7].

The D-loop sequence of the mitochondrial DNA control region analyzed in the ECU population showed high polymorphism (24 haplotypes in total).

The Morona Santiago samples showed greater haplotype diversity than in the Cotopaxi samples, even though the values were low, similar to the value of 0.29 reported in the Zimbabwe chicken [43]. Higher values of 0.515 and 0.908 were found in the Guangxi and Nandan native chickens, respectively [44].

In contrast, the nucleotide diversity was higher in samples from Tungurahua than that in those from Bolívar; samples from Morona Santiago registered a higher value than those from Cotopaxi. Nucleotide diversity is a parameter that estimates the genetic diversity in the population, because this value addresses both the frequency of haplotypes and the differences in nucleotides between haplotypes [16].

Phylogenetic analysis of the haplotypes showed that the Ecuadorian samples clustered in three common maternal lineages: E, F, and A, which are related to the Eurasian region [5,45,46].

Haplogroup E has been observed in the Chilean Araucana chicken [3], which reflects the persistence of ancient Polynesian haplogroups [47]. In the present study, as was previously reported in the Chilean chicken [4], haplotypes belonging to the geographically broad type E were found; this haplogroup has been identified in European animals (Plymouth Rock, White Plymouth Rock, White Leghorn, and New Hampshire), as well as in the Middle East, India, and China [45]. Regarding the haplogroup F, it is mainly concentrated in the southwest of China and in native breeds from Japan, in which Haplogroup F was reported in the Shamo breed from Okinawa [48].

Haplogroup A is distributed all over Eastern Asia [5,44,45]. In birds from Latin America, and more precisely in Chile, haplogroups A, B, and E were also observed, and is the possible path for the introduction of haplogroups A and B, as well as the spread of post-colonial introduced European chickens [4,6], or the introgression of common commercial lineages associated in the three haplogroups A, B, and E1 [5].

The results of the AMOVA did not show a clear divergence between groups; in fact, the percentages of negative variance would be a sign that there is no significant difference between the provinces. This, in relation to mitochondrial DNA, would indicate that all animals belong to the same genetic base. The province of Morona Santiago showed a small, but significant variation, perhaps indicating a strong genetic drift owing to geographical isolation.

The results from the D-loop inference demonstrate that the Ecuadorian chicken originated from at least two different sources. Both linages were identifiable with the European strain and with the Asian type, demonstrating the presence of pre-Columbian influence on the actual population, as was also recently demonstrated in other South American populations, including the Ecuadorian chicken, by other authors [47]. The complete history of the chicken in South America will require the extension of this kind of study to more populations, because the introduction of animals even from the Pacific Ocean could have occurred at different moments and from different starting populations, so our knowledge about mitochondrial DNA diffusion is likely to be changed in the future.

### 4.2. Microsatellites Markers

#### 4.2.1. Genetic Diversity

The mean FIS value of the population was 0.146 (*p* < 0.05) and is similar to that obtained by Suh et al. [49] in local Korean chickens, but lower than what was described by Ding et al. using the same set of markers in native Chinese chickens [50]; other authors describe a lower value of FIS [51,52]. Because of the high significant value of FIS and as a high number of markers deviate from the HWE, it follows that this population is not in equilibrium, probably owing to the existence of a genetic substructure. This situation could be confirmed as the high genetic diversity measured with different parameters does not presuppose erosion or consanguinity phenomena, indicating that the imbalance in HWE is to a large extent because of a non-homogeneous population.

AE is an indicator of genetic variability essential to establish the long-term evolutionary potential of the population, because the selection response is determined by the initial number of alleles [53,54,55]. In the six provinces, high AE levels were presented when compared with those found by Ceccobelli et al. in European breeds [9].

The expected heterozygosity was higher than the observed heterozygosity in the six provinces; therefore, it is suggested that there is no bottleneck effect present in the populations [56]. This last hypothesis could be consistent with the fact that the population in this study is raised in a family environment with little reproductive and genetic planning; therefore, each breed can result in a genetic island with respect to the population (province) to which it belongs. This would also be proven by the fact that a certain decrease in value of FIS index was observed when the calculations were performed with separating the individuals by province instead of treating them as a single population; a behavior described and theorized by different authors [57].

The private alleles are present in greater numbers in differentiated breeds [58]. In the present study, these alleles were consistent with those found by other authors [56,59], although we observed a higher value in the province of Tungurahua, which is genetically more divergent than the other provinces, possibly owing to the receptiveness for intensive poultry farming in this region and some examples of genetic improvement [60,61,62].

When a population has several unbalanced microsatellites, it probably indicates that the population is under some systematic and random forces, which change the genotypic frequencies: mutation, selection, migration, or drift [63]. In the case of the population from Chimborazo, the high imbalance in HWE may be owing to the presence of subzones or parishes with different climatic conditions, which may result in geographical isolation. In Morona Santiago, a lower imbalance could be owing to the extensive local chicken production and, even though a precise breeding strategy does not exist, the selection of future cocks is made empirically by the producers based on their phenotype or market requirement [63]. This allows a certain movement of animals, reducing consanguinity and isolation within the region. The imbalance in the two markers (MCW014, LEI0192) in the six provinces could be attributed to the presence of genotyping errors caused, for example, by null alleles [60].

#### 4.2.2. Genetic Structure

Figure 3 shows the Reynolds distance net constructed by the neighbor-net method. The closer position of ECU to CASN is clear, but it is also close to SUR and UP, highlighting that the ECU chicken could share some common genetic relationship with the Spanish local chicken, demonstrating that the actual population had been strongly influenced by animal introduction during the colonial era. Similarly, the shorter distance with ARAU and the sharing of the central admixture network on the tree demonstrates that a certain common origin of these two Creole types is also because of the relatively shorter geographical distance between Ecuador and Chile. The Spanish breed CASN is closer to ECU, while the LEGH breed is the most distant, confirming the data previously obtained and described in the genetic distance matrix.

The genetic differentiation among the 15 avian populations included in the study was very high, as seen in the F statistic values. The genetic differentiation of the ECU chicken is superior to that found by Francesch et al. in the Penedesenca chicken breed from the Catalonia region of Spain [64].

The values of FST between ECU and some Spanish breeds (CES, IBI, CASN,) or ARAU as well as with CORN, were relatively low if we compare it with previous reports in chickens [59]. This may be because of the fact that in the latter case, well-defined populations were studied with an improvement or conservation program already established, while in our case, similar to the findings of Kong et al. [65], the poor definition of the populations and possible unknown genetic similarities register less pronounced values as well as a stronger genetic relationship. A high value with respect to LEGH makes us suppose poor genetic relationship and an absence of recent crossing with this industrial type, which is not so evident with CORN [66]. This is consistent with the findings of different studies and the AFC results, which indicate the breed with the greatest genetic distance from the Ecuador chicken is the Leghorn [67,68]. These results are similar to those obtained in other studies [59,69,70]. CASN and ARAU showed less genetic distance, as observed in the results.

This may be because the CASN breed was one of the first chickens that Christopher Columbus took to America; the same one that is known as an autochthonous breed of Spain defined at the end of the 19th century. It was very important as a white-shell egg layer during the first half of the 20th century, and it is necessary to note the faneroptic resemblance it has with the animals of the present study. As for the Araucana chicken, its lower genetic distance could be because the geographic location is close to Ecuador, which increases the possibility of crossing, as it is a bird widely distributed in southern Chile. Chickens with some proportion of the Araucana type genome have the characteristics of producing the eggs with blue shells, which is highly valued in Ecuador because the consumer associates this characteristic with a traditional production system [52]. In a study evaluating 15 indigenous breeds from China, the authors observed, using AFC, the formation of a homogeneous cluster comprising the local breeds and a clear distance from LEGH [71].

The results of the Bayesian analysis further confirmed the findings from the previous analysis. From the analysis with the 15 breeds used in the comparative study, we determined that, as expected, there is a strong influence of the Iberian chicken strains in the genome proportions of the Ecuadorian chicken; however, this must be understood based on two aspects. First, there is a strong influence of SUR, because, as already described in our recent investigations in cattle and pigs [72], the breeds of southern Spain were used in the early colonization of South America. Second, the strong relationship between CES and ECU can be because of the presence in Ecuador of a huge population of fighting cocks. Many breeders, who routinely import animals from Spain, where there is an important Fighting cock population [73,74], normally rear these animals in proximity with the Ecuadorian Creole chicken, allowing crossbreeding in many cases. The results with the Evanno method commonly used for the estimation of the most likely number of K was doubtful in this case, although it would seem to indicate a very low K, while from the second method of the graph of the probability already described for the software, there would be a higher value [35]. This behavior had already been noticed by other authors and is probably owing to the strong stratification of domestic poultry populations, as a result of geographical isolation and the lack of breeding programs [9].

In the analysis with STRUCTURE showing only the data for ECU, we see the formation of a cluster for K2 that includes the municipalities of the province of Chimborazo and, unexpectedly, the municipality of Bucay (Guayas). The municipality of Bucay is the gateway to the coastal region of Guayas for the population of the mountain region; a rich province of a commercial nature and home to numerous livestock markets. In fact, the vertical trade of mountain and coastal products is very common in Ecuador; the farmers of the Chimborazo province, in particular, trade potatoes, chickens, cuy (*Cavia porcellus*), and other livestock, while they mainly buy fruit and vegetable products (plane trees, fruit, corn) for the local market in the Andean region. The relationship between these two areas is thus explained by the fact that it is probable that in Bucay, family farmers use genotypes from the mountain for their own production.

In K3, we observe the same subdivision over a further group corresponding to the municipalities of Pelileo and Tisaleo (province of Tungurahua). These two areas of the mountainous region of Ecuador have the greatest concentration of feed industries and, therefore, as in other parts of the world with industrial poultry farms (especially Leghorn), it is logical to assume that local producers have easy access to genetically improved animals of this industrial genotype and use it to improve the performance of the Ecuadorian Creole hens—in particular, to improve the persistence of egg laying. This is a common practice in all the Andean slopes because, in addition to exploiting the rustic qualities of the Ecuadorian Creole hens and the productivity of the Leghorn, the widespread nature of the blue egg is maintained (a genetic character derived from the pre-Columbian Araucana Type hen), and which is now absorbed in the Creole population. Some detail is needed regarding the results of most likely K according to the Evanno method. Several authors noted that misinterpretation or doubtful results in the value of K can be led by the convergence problem of the Gibbs sampler algorithm used in STRUCTURE [75]; the multiple peaks in the Delta K plot may be explained by the existence of a subpopulation [76].

## 5. Conclusions

Ecuadorian chickens reared in the three agroecological systems existing in the country showed a common pattern of haplogroups of mitochondrial DNA, indicating that these animals show the same maternal lineages. The patterns found seem to have the maternal origin in pre-Columbian Asiatic matrilines or in Iberian matrilines arriving from the Iberian Peninsula during colonization. Our mitochondrial findings demonstrate that the current Ecuadorian local chickens do not show maternal influences from commercial lines.

The genetic parameters obtained from our microsatellite findings indicate high levels of genetic diversity; there are no evident effects of genetic drift and/or bottlenecks. This high diversity may be owing to internal heterogeneity, which permits a certain optimism in terms of conservation.

Concerning the origin of the Ecuadorian chicken, microsatellite findings are consistent with that of the mitochondrial DNA. The high proximity to an old Spanish breed such as Castellana Negra, and to the Araucana breed under controversy for its potential origin in Asiatic pre-Columbian branches, demonstrates a double origin (Iberian/Asiatic) of the Ecuadorian chickens. Our study has found a certain internal substructure in the Ecuadorian population, but the absence of any breeding program, registers, or zootechnic management produces a high fragmentation of the potential Ecuadorian breeds. Urgent measures to define, conserve, and manage the genetic diversity of chickens found in Ecuador are necessary.

This study is not only a first step to obtain the genetic characterization of the Ecuadorian chicken breeds, but it is also a model to be followed by other countries in the region, because ignorance regarding the chicken’s Latin American native resources is widespread.

## Figures and Tables

**Table 1:** Mitochondrial DNA diversity indices of the Ecuadorian Creole chicken breed.

Province	n	H	S	Hd	Π	D-Tajima
Bolívar	31	6	5	0.301	0.00091	−2.0081 *
Chimborazo	70	9	84	0.358	0.00745	−2.8870 ***
Guayas	28	7	9	0.442	0.00235	−2.01611 *
Tungurahua	35	4	66	0.311	0.01121	−2.76689 ***
Cotopaxi	32	6	7	0.292	0.00140	−2.07960*
Morona Santiago	38	10	37	0.461	0.00577	−2.70578 ***
All samples	234	24	123	0.359	0.00542	−2.85904 ***

N, number of individuals; H, number of haplotypes; S, polymorphic sites; Hd, haplotype diversity; π, nucleotide diversity. Significance: *p* < 0.05, *; *p* < 0.01, **; *p* < 0.001, ***.

**Table 2:** Genetic diversity parameters of the Ecuadorian chicken breed in each province.

Province	N	He	Ho	NA	AE	PAR	FIS
Bolivar	35	0.6298	0.5500	5.80	3.58	0.25	0.128 *
Chimborazo	72	0.6104	0.5341	6.20	3.46	0.23	0.126 *
Cotopaxi	32	0.6039	0.5251	5.40	3.39	0.19	0.132 *
Guayas	30	0.6531	0.5903	5.50	3.61	0.23	0.098 *
Morona-Santiago	39	0.6426	0.5809	5.77	3.60	0.26	0.097 *
Tungurahua	36	0.6284	0.5425	5.37	3.54	0.31	0.138 *

N, total samples collected in the province; He, expected heterozygosity; Ho, observed heterozygosity; NA, mean number of alleles; AE, effective allele number; PAR, private allele richness; FIS, subpopulation fixation index; *, *p* < 0.05.

## Supplementary Materials

The following are available online at https://www.mdpi.com/2076-2615/9/9/670/s1. Table S1. Municipality where sample have been collected, Latitude (LAT), Longitude (LONG), climate description, and meter above sea level (M, A, S, L). Table S2. Fifteen chicken breeds for population relationship and geneflow assessment. Table S3. Results from AMOVA calculation of D-loop sequence in three different groups representing different starting hypothesis. Table S4. Genetic parameters of microsatellites marker used on the Ecuadorian creole chicken breeds. Mean number of allele (NA), effective allele numbers (AE), expected heterozygosity (He), observed heterozygosity (Ho), polymorphic information content (PIC), fixation index on population (FIS) and its confidence interval (IC), Hardy–Weinberg equilibrium deviation (HW). Table S5. Genetic parameters of microsatellites marker on the 15 chicken population studied. Number of individual (N), mean number of allele (NA), effective allele numbers (AE), expected heterozygosity (He), observed heterozygosity (Ho), fixation index on population (FIS). Table S6. Probability test results for Hardy–Weinberg equilibrium in the six provinces. N^1^: number of marker deviation in a population; N^2^: number of populations deviating for a marker. Table S7. Reynolds genetic distances values (low) and FST pairwise distance (above) between the 15 chicken population included in this study. Table S8. Population membership coefficient of the 15 analysed breeds using the structure software (a) at K11 (around the most likely K according to mean log plot) and 15 (when K = number of population). Gradient indicates lower (darker colour) to higher (lighter colour) membership coefficient. Table S9. Population membership coefficient of the 26 municipalities using the structure software (a) at K2 and 3. Gradient indicates lower (lighter colour) to higher (darker colour) membership coefficient. Figure S1: Map showing the six provinces included in this study and some phenotypes of the collected animals. Figure S2: Factorial correspondence analysis diagrams showing two axes combination. ECU: Ecuadorian; AAZ: Andaluza Azul; CASN: Castellana Negra; CES: Combatiente Español; EAZ: Extremeña Azul; IB: Ibicenca; MLL: Mallorquina; PPA: PitaPinta; SUR: Sureña, UP: Utrerana Perdiz; ARAU: Araucana; BRAH: Brahma; NIG: Nigeria; CORN: Cornish; LEGH: Leghorn. Figure S3. Individual membership coefficient bar plot derived from structure analysis on 15 chicken breeds from K2 to 16. Figure S4. (a) Mean L (K) plot from 10 independent runs over K2 to 15 of the Structure software in 15 chicken breeds included in this study and (b) delta K values plot according Evanno et al (2005). Figure S5. (a) Mean L (K) plot from 10 independent runs over K2 to 27 of the 244 Ecuadorian chicken breeds and (b) delta K values plot according Evanno et al (2005). Figure S6: Sample location according the 26 municipalities belonging to the 6 provinces. Echeandia (1); San Pablo (2); Chimbo (3), Bucay (4), S, Vicente-Cumanda (5), Pallatanga (6), Columbe (7), Nabuzo-Penipe (8), Nabuzo-Penipe (9), Licto (10), Chambo (11), Guano (12), Pelileo (13), Tisaleo (14), Ambato (15), Baños (16), Santa Cecilia (17), Pujili (18), Poalo (19), Belisario (20), Salcedo (21), Saquisili (22), Sevilla Don Bosco (23), Sinai (24), Tres Marias (25), Sevilla De Oro (26).
