# Supplementary material for: Deciphering the Patterns of Genetic Admixture and Diversity in the Ecuadorian Creole Chicken
Source: Animals (Basel). 2019 Sep 11;9(9):670. doi: 10.3390/ani9090670 (PMC6770841; doi:10.3390/ani9090670)
Supplement: Supplementary file 1 [file animals-09-00670-s001.zip › Figure S2.pdf]

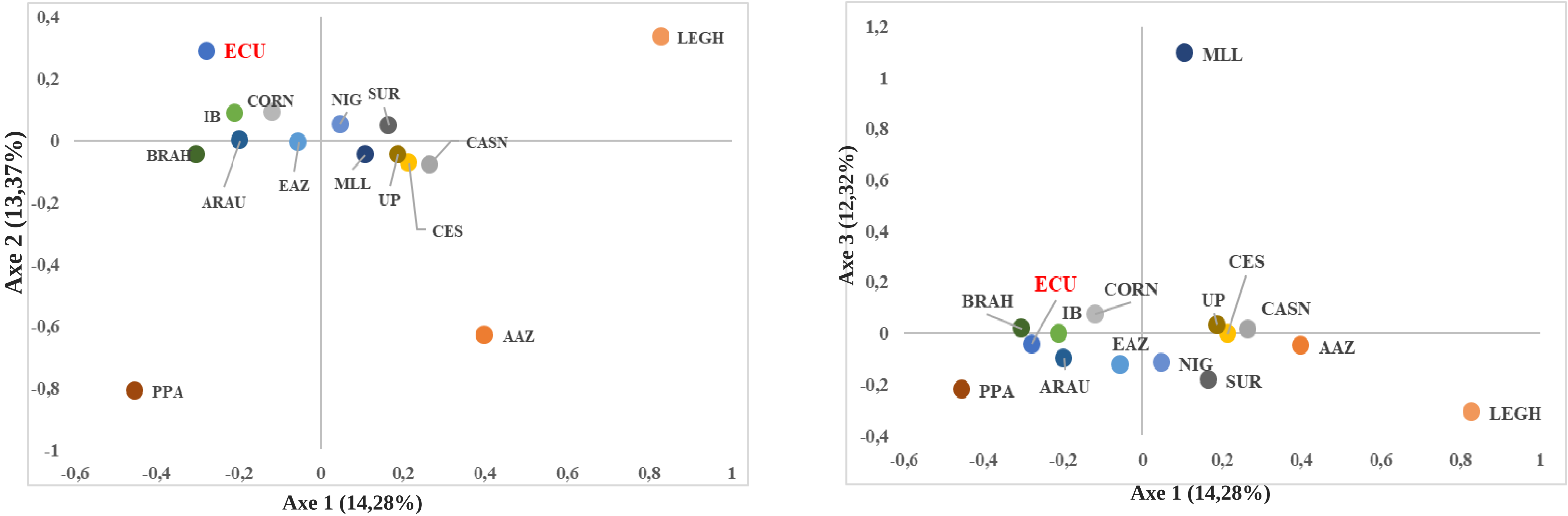

Figure S2: Factorial correspondence analysis diagrams showing 2 axes combination. ECU: Ecuadorian ; AAZ: Andaluza Azul; CASN: Castellana Negra; CES: Combatiente Español; EAZ: Extremeña Azul; IB: Ibicenca; MLL: Mallorquina; PPA: Pita Pinta; SUR: Sureña, UP:Utrerana Perdiz; ARAU: Araucana; BRAH:Brahma; NIG: Nigeria; CORN: Cornish; LEGH: Leghorn
