# Supplementary material for: Deciphering the Patterns of Genetic Admixture and Diversity in the Ecuadorian Creole Chicken
Source: Animals (Basel). 2019 Sep 11;9(9):670. doi: 10.3390/ani9090670 (PMC6770841; doi:10.3390/ani9090670)

Figure S3. Individual membership coefficient bar plot derived from Structure analysis on 15 chicken breeds from K2 to 16.

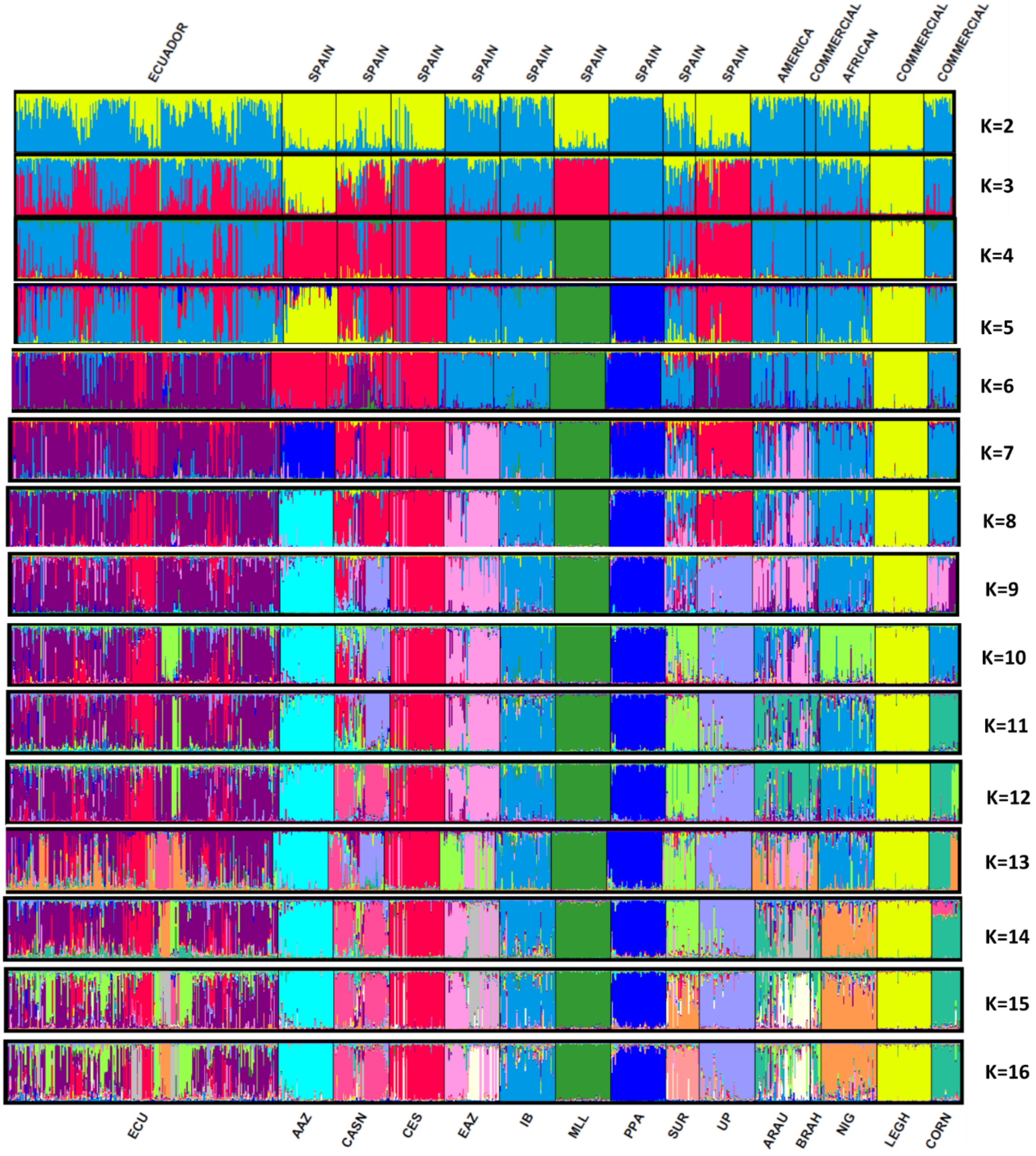

Supplement: Supplementary file 1 [file animals-09-00670-s001.zip › Figure S3.pdf]
