# Supplementary material for: Deciphering the Patterns of Genetic Admixture and Diversity in the Ecuadorian Creole Chicken
Source: Animals (Basel). 2019 Sep 11;9(9):670. doi: 10.3390/ani9090670 (PMC6770841; doi:10.3390/ani9090670)

Figure S4. a) Mean L(K) plot from 10 independent runs over K2 to 15 of the Structure software in 15 chicken breeds included in this study and b) Delta K values plot according Evanno et al (2005)

a)

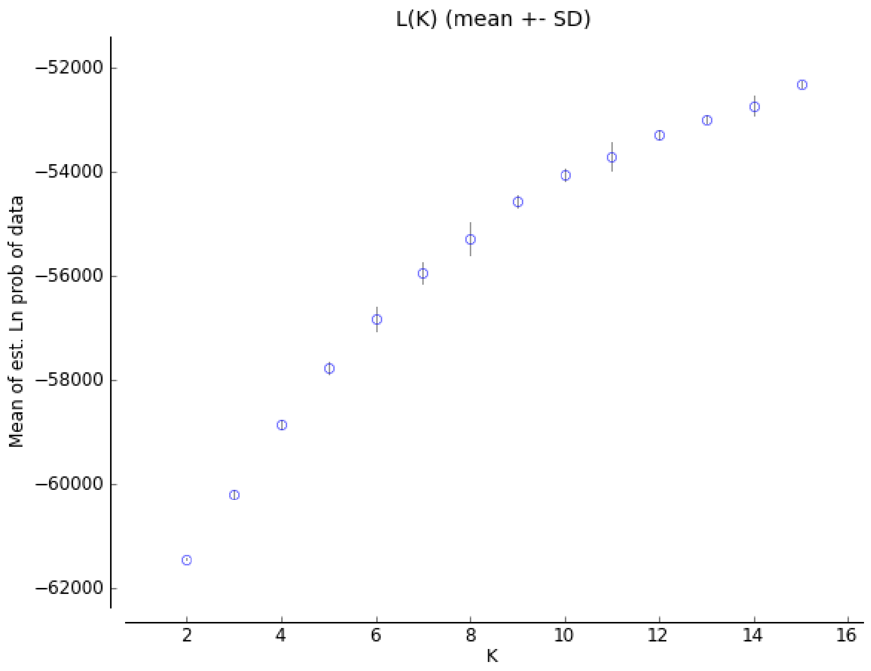

b)

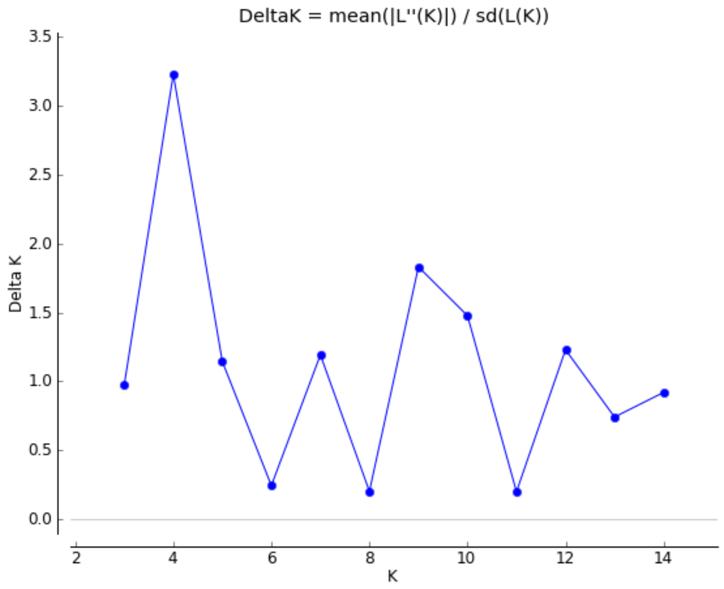

Supplement: Supplementary file 1 [file animals-09-00670-s001.zip › Figure S4 .pdf]
