# Supplementary material for: Deciphering the Patterns of Genetic Admixture and Diversity in the Ecuadorian Creole Chicken
Source: Animals (Basel). 2019 Sep 11;9(9):670. doi: 10.3390/ani9090670 (PMC6770841; doi:10.3390/ani9090670)

Figure S6: Sample location according the 26-municipality belonging to the 6 provinces. Echeandia (1); San Pablo (2); Chimbo (3), Bucay (4), S, Vicente-Cumanda (5), Pallatanga (6), Columbe (7), Nabuzo-Penipe (8), Nabuzo-Penipe (9), Licto (10), Chambo (11), Guano (12), Pelileo (13), Tisaleo (14), Ambato (15), Baños (16), Santa Cecilia (17), Pujili (18), Poalo (19), Belisario (20), Salcedo (21), Saquisili (22), Sevilla Don Bosco (23), Sinai (24), Tres Marias (25), Sevilla De Oro (26)

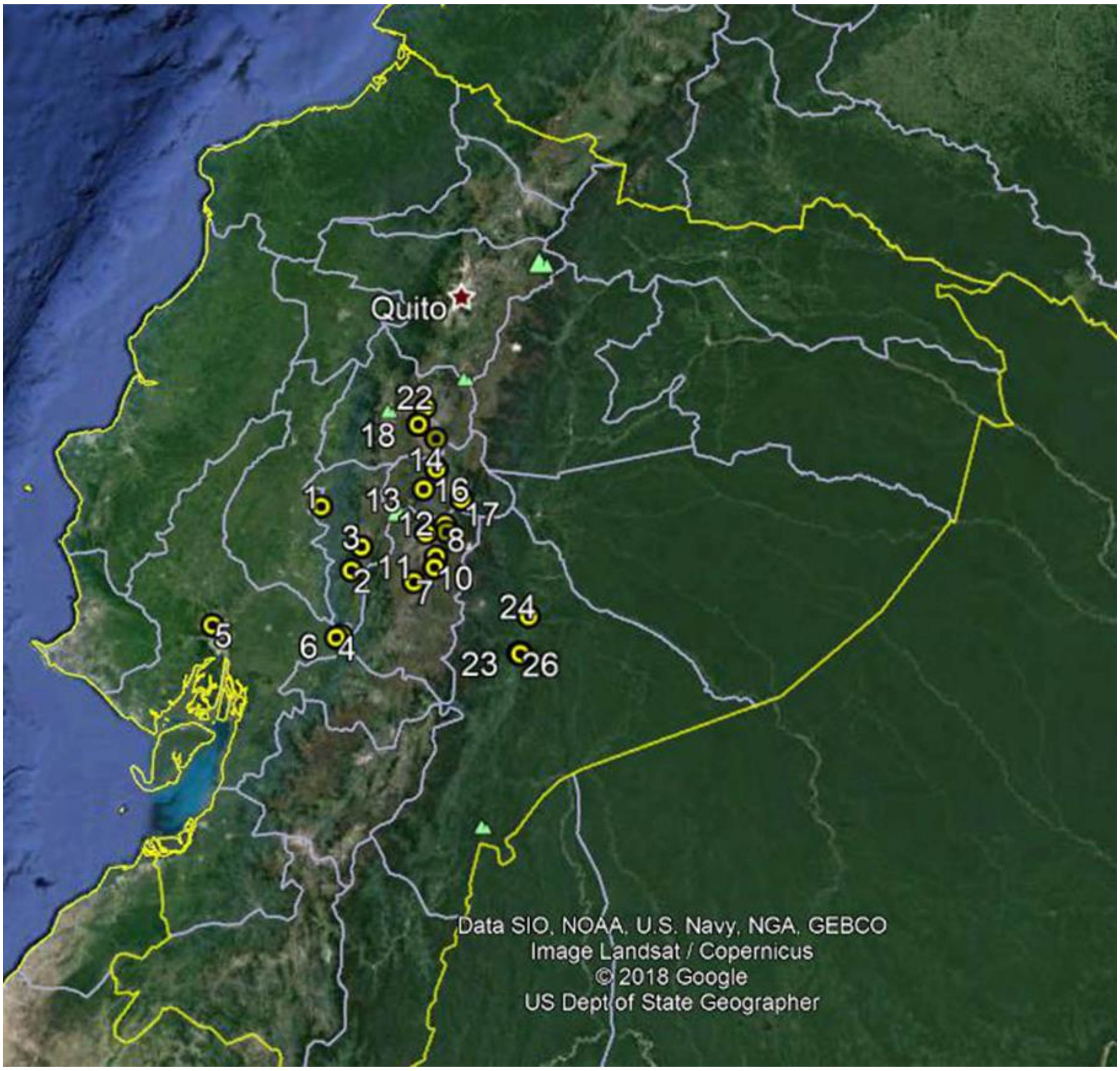

Supplement: Supplementary file 1 [file animals-09-00670-s001.zip › Figure S6.pdf]
