# Supplementary material for: Deciphering the Patterns of Genetic Admixture and Diversity in the Ecuadorian Creole Chicken
Source: Animals (Basel). 2019 Sep 11;9(9):670. doi: 10.3390/ani9090670 (PMC6770841; doi:10.3390/ani9090670)
Supplement: Supplementary file 1 [file animals-09-00670-s001.zip › Tabla S4 edited.docx]

**Table S4.** Genetic parameters of microsatellites marker used on the Ecuadorian Creole chicken breeds. Mean number of allele (NA), effective allele numbers (AE), expected heterozygosity (He), observed heterozygosity (Ho), polymorphic information content (PIC), fixation index on population (FIS) and its confidence interval (IC), and Hardy–Weinberg equilibrium deviation (HW).

| **Marker** | **NA** | **Ae** | **Ho** | **He** | **PIC** | **FIS** | **IC (95%))** | **HW** |
| --- | --- | --- | --- | --- | --- | --- | --- | --- |
| ADL112 | 7 | 4.11 | 0.567 | 0.758 | 0.725 | 0.253 * | (0.1699–0.3327) | *** |
| ADL268 | 6 | 3.9 | 0.643 | 0.745 | 0.701 | 0.136 * | (0.0559–0.2046) | NS |
| ADL278 | 5 | 2.73 | 0.531 | 0.635 | 0.571 | 0.164 * | (0.0699–0.2508) | *** |
| LEI0094 | 14 | 4.76 | 0.736 | 0.792 | 0.761 | 0.071 | (−0.0004–0.1336) | NS |
| LEI0166 | 4 | 2.44 | 0.541 | 0.591 | 0.53 | 0.086 | (−0.0137–0.1897) | *** |
| LEI0192 | 14 | 4.84 | 0.498 | 0.795 | 0.777 | 0.374 * | (0.2860–0.4508) | *** |
| LEI0234 | 19 | 6.67 | 0.788 | 0.852 | 0.839 | 0.075 * | (0.0165–0.1283) | NS |
| MCW014 | 7 | 1.5 | 0.091 | 0.331 | 0.302 | 0.727 * | (0.6178–0.8260) | *** |
| MCW016 | 9 | 3.17 | 0.622 | 0.686 | 0.64 | 0.092 * | (0.0049–0.1739) | NS |
| MCW020 | 4 | 3.32 | 0.634 | 0.7 | 0.65 | 0.095 * | (0.0134–0.1836) | NS |
| MCW034 | 13 | 6.51 | 0.758 | 0.848 | 0.829 | 0.106 * | (0.0450–0.1687) | *** |
| MCW037 | 4 | 2.16 | 0.556 | 0.538 | 0.48 | −0.034 | (−0.1230–0.0521) | NS |
| MCW067 | 6 | 2.9 | 0.622 | 0.656 | 0.587 | 0.052 | (−0.0394–0.1367) | NS |
| MCW069 | 10 | 2.75 | 0.627 | 0.638 | 0.583 | 0.017 | (−0.0687–0.1031) | NS |
| MCW078 | 5 | 2.66 | 0.49 | 0.625 | 0.556 | 0.217 * | (0.1255–0.3113) | *** |
| MCW080 | 11 | 3.54 | 0.661 | 0.719 | 0.685 | 0.081 * | (0.0124–0.1524) | NS |
| MCW081 | 8 | 3.4 | 0.635 | 0.707 | 0.656 | 0.102 * | (0.0245–0.1864) | NS |
| MCW098 | 3 | 1.8 | 0.386 | 0.453 | 0.352 | 0.148 * | (0.0135–0.2748) | NS |
| MCW103 | 3 | 1.7 | 0.402 | 0.41 | 0.33 | 0.02 | (–0.1146–0.1500) | NS |
| MCW104 | 17 | 4.18 | 0.591 | 0.762 | 0.742 | 0.225 * | (0.1495–0.2981) | *** |
| MCW111 | 7 | 3.22 | 0.608 | 0.691 | 0.644 | 0.120 * | (0.0348–0.1972) | NS |
| MCW123 | 8 | 2.5 | 0.517 | 0.601 | 0.557 | 0.141 * | (0.0515–0.2250) | NS |
| MCW165 | 5 | 2.65 | 0.426 | 0.624 | 0.56 | 0.317 * | (0.2173–0.4065) | *** |
| MCW183 | 12 | 2.46 | 0.566 | 0.595 | 0.573 | 0.049 | (−0.0310–0.1276) | NS |
| MCW206 | 11 | 3.8 | 0.631 | 0.736 | 0.703 | 0.143 * | (0.0686–0.2208) | NS |
| MCW216 | 6 | 2.37 | 0.494 | 0.579 | 0.488 | 0.148 * | (0.0490–0.2474) | NS |
| MCW222 | 4 | 1.55 | 0.305 | 0.358 | 0.337 | 0.149 * | (0.0324–0.2706) | *** |
| MCW248 | 4 | 1.56 | 0.35 | 0.359 | 0.325 | 0.026 | (−0.06810–0.1261) | NS |
| MCW295 | 10 | 3.1 | 0.613 | 0.675 | 0.64 | 0.093 * | (0.0134–0.1722) | *** |
| MCW330 | 7 | 2.75 | 0.421 | 0.637 | 0.57 | 0.340 * | (0.2464–0.4245) | *** |
| **Mean** | **8.1** | **3.17** | **0.544** | **0.637** | **0.59** | **0.146** | (**0.1254–0.1638**) | |

*** = *p* < 0.001
