# Supplementary material for: Deciphering the Patterns of Genetic Admixture and Diversity in the Ecuadorian Creole Chicken
Source: Animals (Basel). 2019 Sep 11;9(9):670. doi: 10.3390/ani9090670 (PMC6770841; doi:10.3390/ani9090670)
Supplement: Supplementary file 1 [file animals-09-00670-s001.zip › Tabla S6 edited.docx]

**Table S6.** Probability test results for Hardy–Weinberg equilibrium in the six provinces. N ^1^: number of marker deviation in a population; N ^2^: number of populations deviating for a marker.

| **Province** | **ADL112** | **ADL268** | **ADL278** | **LEI0094** | **LEI0166** | **LEI0192** | **LEI0234** | **MCW014** | **MCW016** | **MCW020** | **MCW034** | **MCW037** | **MCW067** | **MCW069** | **MCW078** | **MCW080** | **MCW081** | **MCW098** | **MCW103** | **MCW104** | **MCW111** | **MCW123** | **MCW165** | **MCW183** | **MCW206** | **MCW216** | **MCW222** | **MCW248** | **MCW295** | **MCW330** | **N ^1^** |
| --- | --- | --- | --- | --- | --- | --- | --- | --- | --- | --- | --- | --- | --- | --- | --- | --- | --- | --- | --- | --- | --- | --- | --- | --- | --- | --- | --- | --- | --- | --- | --- |
| BOL |  |  |  | * | * | ** |  | ** |  |  |  |  |  |  |  |  |  |  |  | ** |  | * | ** |  | ** |  | ** |  |  | * | 10 |
| CHIMB | * | ** | * |  |  | ** |  | ** |  | * | ** |  |  |  | ** |  | * |  |  | ** |  | ** | * |  |  | ** |  |  | * | ** | 15 |
| COT |  |  |  |  |  | ** |  | ** |  |  | * |  | * |  |  | * |  |  |  | ** |  |  |  |  |  |  |  |  |  | ** | 7 |
| GUAY |  |  |  |  |  | ** |  | ** |  |  |  |  |  |  | * |  |  |  | * |  |  |  |  | * |  |  |  |  |  | * | 6 |
| MORO |  |  |  | * |  | ** |  | ** |  |  |  |  |  |  |  |  |  |  |  |  | * |  |  |  |  |  |  |  |  |  | 4 |
| TUNG | ** | ** |  |  | ** | ** | ** | ** |  |  | * |  |  |  | * |  |  |  |  | ** | * | ** | ** |  |  |  |  |  | * | * | 14 |
| N ^2^ | 2 | 2 | 1 | 2 | 2 | 6 | 1 | 6 | 0 | 1 | 3 | 0 | 1 | 0 | 3 | 1 | 1 | 0 | 1 | 4 | 2 | 3 | 3 | 1 | 1 | 1 | 1 | 0 | 2 | 5 |  |

* p < 0.05; ** p < 0.01. Bolívar (BOL), Chimborazo (CHIMB), Cotopaxi (COT), Guayas (GUAY), Morona Santiago (MORO), Tungurahua (TUNG).
