# Supplementary material for: Deciphering the Patterns of Genetic Admixture and Diversity in the Ecuadorian Creole Chicken
Source: Animals (Basel). 2019 Sep 11;9(9):670. doi: 10.3390/ani9090670 (PMC6770841; doi:10.3390/ani9090670)
Supplement: Supplementary file 1 [file animals-09-00670-s001.zip › Table S1 edited.docx]

**Table S1.** Municipality where sample was collected; latitude (LAT), longitude (LONG), climate description and meter above sea level (M, A, S, L).

| **MUNICIPALITY/PROVINCE** | **N** | **LAT** | **LONG** | **CLIMATE** | **M, A, S, L,** |
| --- | --- | --- | --- | --- | --- |
| Echeandia (Bol) | 21 | −1.431431 | −79.280668 | Tropical Mega Thermic Semi-Humid | 327 |
| San Pablo (Bol) | 8 | −1.813655 | −79.092409 | Mega Thermic. Semi-Humid | 2700 |
| Chimbo (Bol) | 6 | −1.676273 | −79.033972 | Mesothermic. Equatorial Semi-Humid | 1500 |
| Bucay (Gua) | 11 | −2.204590 | −79.177840 | Warm and rainy | 700 |
| S, Vicente-Cumanda (Gua) | 13 | −2.120354 | −79.916784 | Sub-Tropical | 1623 |
| Pallatanga (Chi) | 6 | −2.194644 | −79.148234 | Sub-Tropical | 1469 |
| Columbe (Chi) | 22 | −1.888049 | −78.722821 | Alpine | 3341 |
| Nabuzo-Penipe (Chi) | 18 | −1.554858 | −78.541024 | Cold Equatorial. Semi-Humid | 4300 |
| Nabuzo-Penipe (Chi) | 7 | −1.596610 | −78.533277 | Cold Equatorial. Semi-Humid | 4200 |
| Licto (Chi) | 10 | −1.804582 | −78.602199 | Alpine. Cold Equatorial | 2979 |
| Chambo (Chi) | 8 | −1.740657 | −78.593733 | Tempered | 2800 |
| Guano (Chi) | 7 | −1.601677 | −78.654317 | Tempered | 2799 |
| Pelileo (Tun) | 10 | −1.342792 | −78.669326 | Tempered. Sub-Alpine. Glacial | 2500 |
| Tisaleo (Tun) | 8 | −1.223073 | −78.596032 | Tempered and Dry | 3400 |
| Ambato (Tun) | 7 | −1.223073 | −78.596032 | Tempered | 2600 |
| Baños (Tun) | 9 | −1.403790 | −78.447979 | Tropical Rainy | 1820 |
| Santa Cecilia (Cot) | 2 | −1.403790 | −78.447979 | Tropical Rainy | 1900 |
| Pujili (Cot) | 14 | −0.954693 | −78.705583 | Tempered | 2971 |
| Poalo (Cot) | 7 | −0.954693 | −78.705583 | Tempered | 2917 |
| Belisario (Cot) | 1 | −0.954693 | −78.705583 | Humid and Tempered | 2806 |
| Salcedo (Cot) | 7 | −1.041884 | −78.599625 | Cold and Tempered | 2500 |
| Saquisili (Cot) | 3 | −0.850134 | −78.666380 | Tempered | 2938 |
| Sevilla Don Bosco (Ms) | 8 | −2.309314 | −78.094457 | Tropical | 1300 |
| Sinai (Ms) | 10 | −2.095324 | −78.052346 | Warm and Humid | 1199 |
| Tres Marias (Ms) | 10 | −2.307708 | −78.112278 | Warm and Humid | 1030 |
| Sevilla De Oro (Ms) | 11 | −2.312785 | −78.102204 | Warm and Humid | 2347 |

BOL: Bolívar COT: Cotopaxi; GUA: Guayas; CHI: Chimborazo; TUN: Tungurahua; MS: Morona Santiago.
