# Supplementary material for: Deciphering the Patterns of Genetic Admixture and Diversity in the Ecuadorian Creole Chicken
Source: Animals (Basel). 2019 Sep 11;9(9):670. doi: 10.3390/ani9090670 (PMC6770841; doi:10.3390/ani9090670)
Supplement: Supplementary file 1 [file animals-09-00670-s001.zip › Table S2 edited.docx]

**Table S2**. Fifteen chicken breeds for population relationship and geneflow assessment.

| **Breed** | **Abreviation** | **Sample Size** | **Region/Country** |
| --- | --- | --- | --- |
| Ecuador | ECU | 244 | Ecuador |
| Andaluza Azul | AAZ | 50 | Spain |
| Castellana Negra | CASN | 50 | Spain |
| Combatiente Español | CES | 50 | Spain |
| Extremeña Azul | EAZ | 50 | Spain |
| Ibicenca | IB | 50 | Spain |
| Mallorca | MLL | 50 | Spain |
| Pita Pinta | PPA | 50 | Spain |
| Sureña | SUR | 30 | Spain |
| Utrerana Perdiz | UP | 50 | Spain |
| Araucana | ARAU | 50 | Chile |
| Brahma | BRAH | 10 | International |
| Nigerian | NIG | 50 | Nigeria |
| Leghorn | LEGH | 49 | International |
| Cornish | CORN | 26 | International |
