# Supplementary material for: Deciphering the Patterns of Genetic Admixture and Diversity in the Ecuadorian Creole Chicken
Source: Animals (Basel). 2019 Sep 11;9(9):670. doi: 10.3390/ani9090670 (PMC6770841; doi:10.3390/ani9090670)
Supplement: Supplementary file 1 [file animals-09-00670-s001.zip › Table S3 edited.docx]

**Table S3**. Results from AMOVA calculation of D-loop sequence in three different groups representing different starting hypothesis.

| **Groups** | **Variance Component (% de variación)** | | | | |
| --- | --- | --- | --- | --- | --- |
|  | **Within Population** | **Amoung Population** | **Amoung Group** | **F_ST_** | **P** |
| Geographical proximity | 100.63 | −0.45 | −0.19 | −0.00631 | * |
| Grouped according Provinces | 100.59 | −0.59 |  | −0.00593 | * |
| Morona Santiago vs. the other provinces ^a^ | 100.43 | −0.67 | 0.24 | −0.00434 | * |

* = p < 0.05; ^a^: following a posteriori results in microsatellites data, we observed a greater differentiation of this province, so we decided to test it against the rest; geographical proximity group: Bolívar + Guayas vs. Morona Santiago + Chimborazo vs. Cotopaxi + Tungurahua)
