# Supplementary material for: Deciphering the Patterns of Genetic Admixture and Diversity in the Ecuadorian Creole Chicken
Source: Animals (Basel). 2019 Sep 11;9(9):670. doi: 10.3390/ani9090670 (PMC6770841; doi:10.3390/ani9090670)
Supplement: Supplementary file 1 [file animals-09-00670-s001.zip › Table S5 edited.docx]

**Table S5.** Genetic parameters of microsatellites marker on the 15 chicken population studied. Number of individual (N), mean number of allele (NA), effective allele numbers (AE), expected heterozygosity (He), observed heterozygosity (Ho), fixation index on population (FIS). * = p < 0.05

| **Population** | **N** | **NA** | **AE** | **He** | **Ho** | **Fis** |
| --- | --- | --- | --- | --- | --- | --- |
| ECU | 244 | 7.61 | 3.52 | 0.626 | 0.530 | 0.153 * |
| AAZ | 50 | 4.21 | 2.47 | 0.411 | 0.368 | 0.106 * |
| CASN | 50 | 4.93 | 3.04 | 0.545 | 0.481 | 0.118 * |
| CES | 50 | 4.86 | 2.75 | 0.446 | 0.38 | 0.134 * |
| EAZ | 50 | 5.25 | 3.36 | 0.601 | 0.517 | 0.141 * |
| IB | 50 | 5.11 | 3.33 | 0.597 | 0.516 | 0.137 * |
| MLL | 50 | 3.29 | 2.50 | 0.452 | 0.459 | −0.016 |
| PPA | 50 | 4.82 | 3.10 | 0.542 | 0.454 | 0.164 * |
| SUR | 30 | 5.04 | 3.38 | 0.596 | 0.533 | 0.107 * |
| UP | 50 | 3.93 | 2.77 | 0.503 | 0.496 | 0.014 * |
| ARAU | 50 | 6.50 | 3.85 | 0.659 | 0.582 | 0.119 * |
| BRAH | 10 | 4.18 | 3.68 | 0.680 | 0.639 | 0.065 * |
| NIG | 50 | 5.96 | 3.32 | 0.587 | 0.521 | 0.114 * |
| LEGH | 49 | 3.04 | 2.13 | 0.375 | 0.421 | −0.122 * |
| CORN | 26 | 4.64 | 3.19 | 0.563 | 0.465 | 0.177 * |
| Mean ± SD | 57.3 | 4.89 ± 2.62 | 3.09 ± 0.483 | 0.546 ± 0.357 | 0.491 ± 0.014 | 0.113* |
