# Supplementary material for: Deciphering the Patterns of Genetic Admixture and Diversity in the Ecuadorian Creole Chicken
Source: Animals (Basel). 2019 Sep 11;9(9):670. doi: 10.3390/ani9090670 (PMC6770841; doi:10.3390/ani9090670)
Supplement: Supplementary file 1 [file animals-09-00670-s001.zip › Table S7 edited.docx]

**Table S7**. Reynolds genetic distances values (low) and FST pairwise distance (above) between the 15 chicken population included in this study.


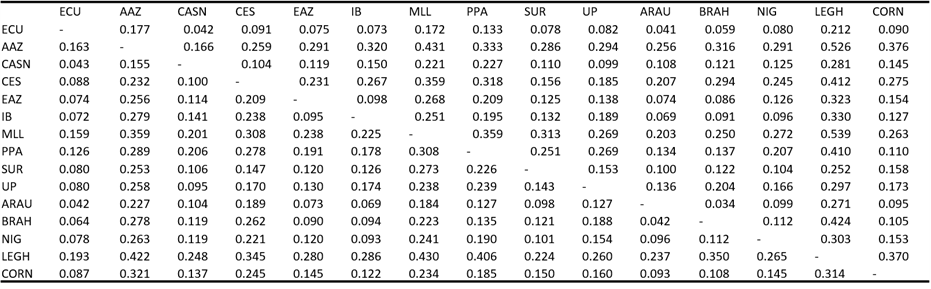


ECU: Ecuadorian; AAZ: Andaluza Azul; CASN: Castellana Negra; CES: Combatiente Español; EAZ: Extremeña Azul; IB: Ibicenca; MLL: Mallorquina; PPA: Pita Pinta; SUR: Sureña, UP: Utrerana Perdiz; ARAU: Araucana; BRAH: Brahma; NIG: Nigeria; CORN: Cornish; LEGH: Leghorn.
