# Supplementary material for: Deciphering the Patterns of Genetic Admixture and Diversity in the Ecuadorian Creole Chicken
Source: Animals (Basel). 2019 Sep 11;9(9):670. doi: 10.3390/ani9090670 (PMC6770841; doi:10.3390/ani9090670)
Supplement: Supplementary file 1 [file animals-09-00670-s001.zip › Table S8 edited.docx]

**Table S8**. Population membership coefficient of the 15 analysed breeds using the Structure software (a) at K11 (around the most likely K according to mean log plot) and 15 (when K = number of population). Gradient indicates lower (darker colour) to higher (lighter colour) membership coefficient.

a)

| **Population** | **1** | **2** | **3** | **4** | **5** | **6** | **7** | **8** | **9** | **10** | **11** |
| --- | --- | --- | --- | --- | --- | --- | --- | --- | --- | --- | --- |
| ECU | 0.15 | 0.027 | 0.019 | 0.112 | 0.035 | 0.026 | 0.01 | 0.016 | 0.021 | 0.57 | 0.014 |
| AAZ | 0.011 | 0.009 | 0.006 | 0.013 | 0.006 | 0.016 | 0.003 | 0.004 | 0.02 | 0.006 | 0.906 |
| CASN | 0.201 | 0.035 | 0.014 | 0.093 | 0.414 | 0.051 | 0.033 | 0.013 | 0.019 | 0.047 | 0.081 |
| CES | 0.849 | 0.005 | 0.007 | 0.019 | 0.01 | 0.012 | 0.007 | 0.008 | 0.065 | 0.011 | 0.008 |
| EAZ | 0.021 | 0.018 | 0.006 | 0.015 | 0.02 | 0.025 | 0.007 | 0.01 | 0.824 | 0.047 | 0.008 |
| IB | 0.008 | 0.784 | 0.012 | 0.009 | 0.016 | 0.042 | 0.025 | 0.036 | 0.037 | 0.023 | 0.008 |
| MLL | 0.004 | 0.004 | 0.003 | 0.003 | 0.004 | 0.004 | 0.004 | 0.962 | 0.004 | 0.004 | 0.004 |
| PPA | 0.006 | 0.007 | 0.914 | 0.009 | 0.011 | 0.011 | 0.006 | 0.005 | 0.011 | 0.011 | 0.01 |
| SUR | 0.031 | 0.027 | 0.008 | 0.725 | 0.027 | 0.064 | 0.025 | 0.009 | 0.063 | 0.011 | 0.01 |
| UP | 0.013 | 0.007 | 0.005 | 0.056 | 0.867 | 0.007 | 0.011 | 0.006 | 0.009 | 0.007 | 0.012 |
| ARAU | 0.014 | 0.072 | 0.041 | 0.023 | 0.029 | 0.544 | 0.014 | 0.02 | 0.136 | 0.094 | 0.012 |
| BRAH | 0.006 | 0.087 | 0.045 | 0.085 | 0.017 | 0.44 | 0.009 | 0.027 | 0.135 | 0.101 | 0.05 |
| NIG | 0.016 | 0.712 | 0.023 | 0.076 | 0.036 | 0.024 | 0.028 | 0.012 | 0.017 | 0.036 | 0.018 |
| LEGH | 0.004 | 0.003 | 0.002 | 0.004 | 0.004 | 0.006 | 0.963 | 0.003 | 0.002 | 0.004 | 0.004 |
| CORN | 0.01 | 0.013 | 0.006 | 0.006 | 0.011 | 0.911 | 0.007 | 0.012 | 0.006 | 0.014 | 0.004 |

b)

| **Population** | **1** | **2** | **3** | **4** | **5** | **6** | **7** | **8** | **9** | **10** | **11** | **12** | **13** | **14** | **15** |
| --- | --- | --- | --- | --- | --- | --- | --- | --- | --- | --- | --- | --- | --- | --- | --- |
| ECU | 0.013 | 0.024 | 0.013 | 0.391 | 0.088 | 0.009 | 0.029 | 0.167 | 0.02 | 0.016 | 0.017 | 0.143 | 0.034 | 0.019 | 0.017 |
| AAZ | 0.896 | 0.007 | 0.015 | 0.004 | 0.007 | 0.003 | 0.011 | 0.009 | 0.01 | 0.004 | 0.01 | 0.009 | 0.004 | 0.006 | 0.005 |
| CASN | 0.038 | 0.022 | 0.022 | 0.026 | 0.046 | 0.019 | 0.021 | 0.013 | 0.696 | 0.007 | 0.02 | 0.024 | 0.029 | 0.006 | 0.01 |
| CES | 0.009 | 0.007 | 0.086 | 0.007 | 0.01 | 0.007 | 0.004 | 0.005 | 0.011 | 0.005 | 0.007 | 0.823 | 0.011 | 0.003 | 0.005 |
| EAZ | 0.008 | 0.009 | 0.048 | 0.028 | 0.01 | 0.007 | 0.021 | 0.02 | 0.01 | 0.008 | 0.787 | 0.017 | 0.013 | 0.009 | 0.006 |
| IB | 0.007 | 0.019 | 0.027 | 0.027 | 0.009 | 0.024 | 0.024 | 0.011 | 0.022 | 0.031 | 0.024 | 0.009 | 0.016 | 0.745 | 0.008 |
| MLL | 0.004 | 0.003 | 0.003 | 0.004 | 0.003 | 0.003 | 0.004 | 0.004 | 0.004 | 0.95 | 0.004 | 0.004 | 0.004 | 0.004 | 0.003 |
| PPA | 0.01 | 0.01 | 0.016 | 0.008 | 0.006 | 0.005 | 0.007 | 0.011 | 0.007 | 0.005 | 0.006 | 0.005 | 0.01 | 0.005 | 0.889 |
| SUR | 0.014 | 0.53 | 0.14 | 0.011 | 0.015 | 0.035 | 0.021 | 0.045 | 0.031 | 0.01 | 0.013 | 0.069 | 0.043 | 0.015 | 0.008 |
| UP | 0.012 | 0.009 | 0.009 | 0.007 | 0.008 | 0.01 | 0.008 | 0.011 | 0.02 | 0.006 | 0.009 | 0.014 | 0.865 | 0.009 | 0.005 |
| ARAU | 0.008 | 0.021 | 0.43 | 0.043 | 0.018 | 0.014 | 0.107 | 0.185 | 0.024 | 0.019 | 0.03 | 0.01 | 0.023 | 0.049 | 0.019 |
| BRAH | 0.032 | 0.055 | 0.284 | 0.023 | 0.01 | 0.01 | 0.118 | 0.286 | 0.056 | 0.025 | 0.02 | 0.004 | 0.007 | 0.049 | 0.021 |
| NIG | 0.012 | 0.746 | 0.017 | 0.035 | 0.025 | 0.011 | 0.023 | 0.019 | 0.014 | 0.012 | 0.023 | 0.008 | 0.014 | 0.022 | 0.018 |
| LEGH | 0.004 | 0.003 | 0.003 | 0.004 | 0.003 | 0.954 | 0.006 | 0.003 | 0.005 | 0.003 | 0.002 | 0.003 | 0.004 | 0.002 | 0.002 |
| CORN | 0.004 | 0.006 | 0.012 | 0.006 | 0.005 | 0.006 | 0.718 | 0.201 | 0.006 | 0.009 | 0.004 | 0.005 | 0.005 | 0.009 | 0.005 |
