# Supplementary material for: Deciphering the Patterns of Genetic Admixture and Diversity in the Ecuadorian Creole Chicken
Source: Animals (Basel). 2019 Sep 11;9(9):670. doi: 10.3390/ani9090670 (PMC6770841; doi:10.3390/ani9090670)
Supplement: Supplementary file 1 [file animals-09-00670-s001.zip › Table S9 edited.docx]

**Table S9** Population membership coefficient of the 26 municipalities using the structure software (a) at K2 and 3. Gradient indicates lower (lighter colour) to higher (darker colour) membership coefficient.

| **Municipality** | **K2** | | **K3** | | |
| --- | --- | --- | --- | --- | --- |
| Echeandia | 0,278 | 0,722 | 0,208 | 0,002 | 0,79 |
| San Pablo | 0,138 | 0,862 | 0,058 | 0,01 | 0,932 |
| Chimbo | 0,11 | 0,89 | 0,042 | 0,021 | 0,938 |
| Columbe | 0,119 | 0,881 | 0,031 | 0,005 | 0,964 |
| Bucay | 0,794 | 0,206 | 0,76 | 0,003 | 0,237 |
| San Vicente-Cumanda | 0,343 | 0,657 | 0,306 | 0,002 | 0,692 |
| Pallatanga | 0,098 | 0,902 | 0,045 | 0,023 | 0,932 |
| Nabuzo-Penipe | 0,132 | 0,868 | 0,039 | 0 | 0,96 |
| Penipe-Nabuzo | 0,761 | 0,239 | 0,738 | 0,004 | 0,258 |
| Licto | 0,965 | 0,035 | 0,95 | 0,001 | 0,048 |
| Chambo | 0,948 | 0,052 | 0,917 | 0,002 | 0,081 |
| Guano | 0,294 | 0,706 | 0,247 | 0,002 | 0,75 |
| Pelileo | 0,01 | 0,99 | 0,01 | 0,758 | 0,232 |
| Tisaleo | 0,035 | 0,965 | 0,02 | 0,007 | 0,973 |
| Ambato | 0,253 | 0,747 | 0,151 | 0,019 | 0,83 |
| Baños | 0,019 | 0,981 | 0,008 | 0,007 | 0,985 |
| Santa Cecilia | 0,105 | 0,895 | 0,034 | 0,014 | 0,952 |
| Pujili | 0,295 | 0,705 | 0,245 | 0,022 | 0,733 |
| Poalo | 0,33 | 0,67 | 0,187 | 0,002 | 0,811 |
| Belisario | 0,713 | 0,287 | 0,731 | 0,035 | 0,234 |
| Salcedo | 0,385 | 0,615 | 0,331 | 0,003 | 0,666 |
| Saquisili | 0,211 | 0,789 | 0,081 | 0,002 | 0,916 |
| Sevilla Don Bosco | 0,031 | 0,969 | 0,018 | 0,005 | 0,977 |
| Sinai | 0,085 | 0,915 | 0,009 | 0 | 0,991 |
| 3 Marias | 0,033 | 0,967 | 0,015 | 0,013 | 0,972 |
| Sevilla De Oro | 0,121 | 0,879 | 0,056 | 0,004 | 0,94 |
